# Managed European-Derived Honey Bee, *Apis mellifera* sspp, Colonies Reduce African-Matriline Honey Bee, *A*. *m*. *scutellata*, Drones at Regional Mating Congregations

**DOI:** 10.1371/journal.pone.0161331

**Published:** 2016-08-12

**Authors:** Ashley N. Mortensen, James D. Ellis

**Affiliations:** Department of Entomology & Nematology, University of Florida, Gainesville, Florida, United States of America; Universidade Federal de Vicosa, BRAZIL

## Abstract

African honey bees (*Apis mellifera scutellata)* dramatically changed the South American beekeeping industry as they rapidly spread through the Americas following their introduction into Brazil. In the present study, we aimed to determine if the management of European-derived honey bees (*A*. *mellifera* sspp.) could reduce the relative abundance of African-matriline drones at regional mating sites known as drone congregation areas (DCAs). We collected 2,400 drones at six DCAs either 0.25 km or >2.8 km from managed European-derived honey bee apiaries. The maternal ancestry of each drone was determined by *Bgl* II enzyme digestion of an amplified portion of the mitochondrial Cytochrome *b* gene. Furthermore, sibship reconstruction via nuclear microsatellites was conducted for a subset of 1,200 drones to estimate the number of colonies contributing drones to each DCA. Results indicate that DCAs distant to managed European apiaries (>2.8 km) had significantly more African−matriline drones (34.33% of the collected drones had African mitochondrial DNA) than did DCAs close (0.25 km) to managed European apiaries (1.83% of the collected drones had African mitochondrial DNA). Furthermore, nuclear sibship reconstruction demonstrated that the reduction in the proportion of African matriline drones at DCAs near apiaries was not simply an increase in the number of European matriline drones at the DCAs but also the result of fewer African matriline colonies contributing drones to the DCAs. Our data demonstrate that the management of European honey bee colonies can dramatically influence the proportion of drones with African matrilines at nearby drone congregation areas, and would likely decreasing the probability that virgin European queens will mate with African drones at those drone congregation areas.

## Introduction

African honey bees, *Apis mellifera scutellata* Lepeletier (1836), are native to semi-arid areas in sub-Saharan Africa. African-derived honey bees rapidly spread through the Americas following their introduction into Brazil in the 1950s [1,2]. Despite the efforts of beekeepers and local agencies to conserve European−derived honey bees (including *A*. *m*. *ligustica* Spinola (1806), *carnica* Pollmann (1879), *mellifera* Linnaeus (1758), *caucasica* Pollmann (1889), and *iberiensis* Engel (1999) [3,4]), African-derived honey bees now predominate the American tropics [5,6]. African-derived honey bees in the Americas are genetically distinguishable from their ancestral population in Africa [7]. However, for conciseness, we will refer to both African- and European-derived honey bees in the Americas as African and European honey bees, respectively.

In South and Central America, managed African honey bees quickly become the predominate subspecies upon entering new territory [8]. African honey bees are better adapted to the tropical environment of South and Central America than are European honey bees and they outperform European honey bees in terms of survivability and honey production there [9]. However, the heightened defensive behavior expressed by African honey bees has dramatically changed the South American beekeeping industry. For example, beekeepers relocated their apiaries into remote, isolated locations and hobbyist beekeeping has become nearly nonexistent [8].

In contrast, African bees are considered undesirable by beekeepers bees in temperate climates for a number of reasons. First, African honey bees are less likely to outperform temperate adapted European bee races in temperate climates [10]. For example, European honey bees perform well in high population densities where food is the limiting resource [11], while African honey bees tend to abscond to areas with plentiful resources [8]. The portion of the bee population with more European ancestry seems favored over that with an African ancestry when competition for limited resources arises [12]. Moreover, the African honey bee’s elevated defensive behavior and high propensity to swarm/abscond make African honey bees undesirable to many beekeepers [13] and a potential safety concern for animals and humans. Therefore, beekeepers managing European bees in areas where African bees are present alter their management practices to limit the integration of African genetics into their managed bees.

One strategy used by beekeepers trying to limit the impact of African bees in the managed European bee population is saturating the environment with European colonies. Beekeepers believe this saturation can modify the regional reproductive population so that European queens are more likely to mate with European drones rather than hybridize with African drones, though this has not been tested directly.

Honey bee mating occurs at distinct locations called drone congregation areas (DCAs [14]). Large numbers of drones aggregate daily at DCAs between 10–30 m above the ground with the intent to mate with a queen [15]. Drones typically do not travel far to a DCA. The majority of drones attend a DCA < 0.5 km from their colony and very few travel > 2.0 km to a DCA [16]. “Africanization” of managed European honey bee colonies occurs through paternal introgression of African genetics (i.e. a European queen mates with an African drone [5]). Therefore, the ability to modify the proportion of African drones at a DCA could reduce the likelihood of Africanized colonies in managed apiaries.

In Chiapas, Mexico, Loper et al. [17] simultaneously employed three techniques to manipulate the reproductive population: 1) trapping and removal of African drones prior to introducing European drone-source colonies; 2) introduction of large numbers of European drone-source colonies to flood the regional population; and 3) strategic placement of those drone-source colonies. However, they were unable to ascertain which factor was most impactful due to the experimental design.

In this study, we aimed to determine if the management of European honey bees could decrease the proportion of African drones present at nearby drone congregations areas. We expected that the management of European colonies would result in proportionally more European drones, and correspondingly fewer African drones, at nearby DCAs.

## Materials and Methods

### Sample Collection

The northern expansion of the African honey bee population in the Southeastern United States has been stable in central Florida since 2005 [18]. Six DCAs were identified within the African honey bee range in Orange and Osceola counties, Florida per [19]. Three of the six DCAs were located within 0.25 km of 96, ten-frame, commercial European colonies kept in Langstroth-style hives. The three remaining DCAs were located > 2.8 km from any managed colonies. Since drones typically travel < 2.0 km to a DCA [20], drones trapped>2.8 km from managed colonies were considered representative of the feral population.

Drones were collected through the duration of the flight time using a Williams [21] drone trap equipped with a queen lure constructed of a 2 cm piece braided dental roll died black with India ink. The queen lure was baited with 1 mg of synthetic 9−oda and suspended 20 cm below the trap opening [22–24]. The Williams trap was attached 5 m below a white 1.2 m chloroprene balloon. The height of the trap was defined as the distance from the bottom of the trap opening to the ground [19]. A minimum of 400 drones was collected from each of the six DCAs. Within each DCA, drones were caught at two heights: a minimum of 200 drones from 10 m above the ground and a minimum of 200 drones from 30 m above the ground. Drones were preserved in 95% ethanol and transported to the laboratory where they were stored at −80°C until molecular processing.

### Mitochindrial (mtDNA) Analysis

Four hundred drones (200 drones from 10 m above the ground and 200 drones from 30 m above the ground) were randomly selected from the total drones collected from each DCA for mtDNA analysis. Total DNA was extracted from a hind leg of each drone using 10% Chelex [25]. Maternal ancestry was determined for each drone using PCR-RFLP for a diagnostic portion of the mitochondrial Cytochrome *b* gene [26]. A 485−bp fragment of the cytochrome *b* gene was amplified by the PCR then digested by *Bgl* II restriction enzymes as described by Pinto et al. [27]. European cytochrome *b* fragments contain the *Bgl* II restriction site and are cleaved into 291− and 194−bp fragments. African cytochrome *b* fragments lack the *Bgl* II restriction site and remain intact (485−bp) [27]. The PCR−RFLP products were visualized on 2.0% agarose gels to identify cleaved (European) or intact (African) mtDNA fragments for each drone.

### Microsatellite Analysis

Subsets of 200 drones were selected from each DCA for nuclear DNA analysis (1200 drones total). Ten microsatellite loci (HB−THE−03, HB−THE−04, HB−C16−05, HB−SEX−01, HB−SEX−03, A7, A79, A88, A107 and B124 [12, 28, 29]) were amplified via PCR (Table 1) from the total DNA extracted for the mtDNA analysis. All forward primers were fluorescently labeled with FAM, VIC, PET, or NED, and all reverse primers were pigtailed (addition of GCTTCT [30]) to minimize stutter on the fragment length results. Individual 10 μl PCR reactions were run for one or two loci at a time. The decision to plex loci for PCR reactions was based on the fluorescent label of the forward primer for those loci and the previously published fragment length range of the loci. Only loci with the same fluorescent label and distinct fragment length ranges were plexed for PCR reactions. The PCR conditions used for all microsatellite reactions are described by Shaibi et al. [29]. The PCR products from each reaction were then diluted by the ratios described in Table 1 and combined into the two analyses plexes. The final plexes were sent to the University of Florida’s Interdisciplinary Center for Biotechnology Research and fragment lengths were analyzed with a 3730xl DNA Analyzer (Applied Biosystems).

**Table 1:** Detailed information on the primers and plexes used for nuclear microsatellite analysis. All PCR reaction were 10 μl total volume. Data are the loci that were grouped for PCR reactions (Reaction Plex); names of the microsatellite (Loci Name); the previously published fragment length of the amplified microsatellites (Fragement Length Ranges); Primer sequence information (including the fluorescent label of the forward primer and the pigtail sequence on the reverse primer (highlighted in bold), Primers); the molar concentration of each primer in each PCR reaction (Primer Concentration); the post reaction plexing groups for analysis; and the dilution ratio for each reaction plex prior to generating the analysis plex (Dilution for Analysis).

Reaction Plex	Loci Name	Fragment Length Ranges	Primers (Fluorescent Label-Forward/(PIGTAIL)Reverse)	Primer Concentration (μM)	Analysis Plex	Dilution for Analysis	References
1	HB−C16−05	65−89	**FAM**−ATTTTATGCGCGTTTCGTA	0.5	1	25:1	29
			**GCTTCT**CATGGCTCCTCCATTAAATC	0.5			
	A107	140−194	**FAM**−CCGTGGGAGGTTTATTGTCG	0.12			29, 12
			**GCTTCT**CCTTCGTAACGGATGACACC	0.12			
2	HB−THE−03	174−209	**NED–**TAACTGGTCGTCGGTGTT	0.18		300:1	29
			**GCTTCT**CACGTAGAGAATCCCATTGT	0.18			
	B124	212−262	**NED–**GCAACAGGTCGGGTTAGAG	0.28			28
			**GCTTCT**CAGGATAGGGTAGGTAAGCAG	0.28			
3	HB−SEX−03	154−210	**FAM**−AACGTGGAAGATAACTTTAACAA	0.48	2	25:1	29
			**GCTTCT**ACAATGTTATGATTTTTCACGA	0.48			
	A79	89−127	**FAM–**CGAAGGTTGCGGAGTCCTC	0.16			12,28,29
			**GCTTCT**GTCGTCGGACCGATGCG	0.16			
4	HB−SEX−01	142−165	**VIC–**AGTGCAAAATCCAAATCATC	0.44		100:1	28
			**GCTTCT**ATTCGATCACCCAAAGAA	0.44			
5	A7	95−172	**PET–**GTTAGTGCCCTCCTCTTGC	0.68		100:1	12,28,29
			**GCTTCT**CCCTTCCTCTTTCATCTTCC	0.68			
6	HB−THE−04	225−239	**NED–**GCTGGAAGGGAACTGTAGA	0.26		200:1	29
			**GCTTCT**GGACGCGTTTTAATATCTCA	0.26			
	A88	136−159	**NED–**CGAATTAACCGATTTGTCG	0.18			12,28
			**GCTTCT**GATCGCAATTATTGAAGGAG	0.18			

Microsatellite fragment lengths were scored using GeneMarker© 2.4.0 (SoftGenetics). Any individuals with failed amplification at more than two of the ten loci were excluded from statistical analysis. There were two linkage groups represented in the 10 microsatellites analyzed, HB−THE-−03 & HB−THE−04 and HB−SEX−01 & HB−SEX−03. Haplotypes were manually assigned to individual drones for each linkage group [31]. Sibship reconstruction was run by COLONY© 2.0.6.1 (ZSL Institute of Zoology) to estimate the number of colonies that had produced the drones present at each DCA [31,32,33,34].

### Statistical Analysis

The proportion of both maternal ancestries (European and African) was compared for drones from each DCA by the proximity of the DCA to managed European honey bee colonies (near (0.25 km) or distant (> 2.8 km)) and height of the DCA from the ground (10 or 30 m) using Pearson’s χ^2^ test in JMP® 11.0.0 (SAS Institute Inc).

Counts of the number of colonies that had produced the drones present at each DCA were log transformed and compared via one-way ANOVAs based on the proximity of the DCA to managed European honey bee colonies (near (0.25 km) or distant (> 2.8 km)). Additionally, mtDNA results were combined with nuclear sibship data to determine the number of colonies that had African mtDNA and had produced the drones present at each DCA. The counts of colonies with African mtDNA were transformed by logging the count + 0.5. One−way ANOVA was run on the transformed African mtDNA colony data based on the proximity of the DCA to managed European honey bee colonies (near (0.25 km) or distant (> 2.8 km)). Lastly, the proportion of the total colonies detected that had African mtDNA was calculated and compared via one-way ANOVAs based on the proximity of the DCA to managed European honey bee colonies (near (0.25 km) or distant (> 2.8 km)). All drones used in the nuclear analysis came form the 10 m elevation sampling. Therefore, vertical height within the DCA was not included in the nuclear DNA analysis as it was in the mtDNA analysis.

### Ethics Statement

All DCAs were located on private property, and permission for sampling was granted by the property owner. No formal permits were required for field collections or laboratory analysis because honey bee research is not regulated by animal use committees such as the Institutional Animal Care and Use Committee (IACUC).

## Results

Mitochondrial DNA analysis results indicate that DCAs distant to managed European honey bee apiaries (> 2.8 km) had significantly more (χ^2^_1, 2400_ = 427.83, *p* <0.0001) African matriline drones (34.33% of the collected individuals) present than did DCAs located close to managed European honey bee apiaries (1.83% of the collected individuals; Table 2). There was no detectible difference in the vertical distribution of African or European drones within DCAs (χ^2^_1, 2400_ = 0.11, *p* = 0.74).

**Table 2:** Mitochondrial DNA results for drones trapped at DCAs within 0.25 km of managed colonies of European honey bees or > 2.8 km from any managed colonies, and proportions of African and European matriline drones present at each DCA location type.

DCA locations	total no. drones analyzed	no. drones with European mtDNA	no. drones with African mtDNA	% of drones with European mtDNA	% of drones with African mtDNA
0.25 km from apiary	400	399	1	99.75%	0.25%
	400	397	3	99.25%	0.75%
	400	382	18	95.50%	4.50%
>2.8 km from apiary	400	231	169	57.75%	42.25%
	400	291	109	72.75%	27.25%
	400	266	134	66.50%	33.50%

Note: The total proportion of drones with African (and correspondingly European) mtDNA was significantly different (χ^2^_1, 2400_ = 427.83, *p* <0.0001) between DCAs near (0.25 km, 1.8% of drones had African mtDNA) and far (>2.8 km, 34.3% of drones had African mtDNA) from managed European honey bee colonies based on Pearson’s χ^2^ test.

Nuclear microsatellite analysis indicates that DCAs near to managed European honey bee apiaries (0.25 km) had significantly more (F = 45.17; df = 1,5; *p* = 0.0026) colonies contributing drones than did DCAs distant to managed European honey bee apiaries (> 2.8 km; Table 3). Additionally, significantly more (F = 45.2364; df = 1,5; *p* = 0.0025) of the colonies contributing drones to DCAs distant to managed European honey bee apiaries (> 2.8 km) had African mtDNA than did the colonies contributing drones to DCAs near to managed European honey bee apiaries (0.25 km). Furthermore, we saw a significant (F = 11.90; df = 1,5; *p* = 0.0261) reduction in the proportion of colonies with African mtDNA that were contributing drones to DCAs near to managed European honey bee apiaries.

**Table 3:** Maternal group results based on the nuclear microsatellite analysis of drones trapped at DCAs within 0.25 km of managed colonies of European honey bees or >2.8 km from any managed colonies, and proportions of those maternal groups that had African mtDNA.

DCA locations	total no. of maternal groups	no. maternal groups with African mtDNA	% of maternal groups with African mtDNA
0.25 km from apiary	58	6	10.34%
	58	0	0.00%
	62	0	0.00%
>2.8 km from apiary	43	25	58.14%
	41	11	26.83%
	47	15	31.91%

## Discussion

Our data demonstrate that the management of European honey bee colonies can influence the proportion of drones with African matrilines at nearby DCAs dramatically. Furthermore, our nuclear analysis confirms that the observed decrease in African matrilines at DCAs near managed apiaries is not merely the result of a large number of managed European colonies producing drones that flood the proximal DCAs. Rather we see an increase in the total number of colonies contributing drones and a decrease in the total number of African matriline colonies contributing drone s to DCAs near managed apiaries. Cumulatively, these changes in population structure lead to a drastic reduction in the presence of African matriline drones at DCAs that are near by managed apiaries.

Our results suggest that the management of European honey bees could be a viable option for limiting the introgression of African bee genetics into European colonies. Managed European colonies become “Africanized” when virgin European queens mate with African drones [5]. Increasing the proportion of European drones present at a DCA is expected to increase the likelihood that virgin queens at that DCA will mate with European, opposed to African, drones [17]. By reducing the likelihood that European queens mate with African drones, the frequency and degree of Africanization in managed apiaries would be reduced.

Virgin queens fly to more distant DCAs than the proximal DCAs that drones from the same apiary attend. Drones typically travel less than 0.5 km to a DCA whereas queens are estimated to travel 2.0 km to a DCA [20, 35]. Therefore, altering the proportion of drones at DCAs nearby an apiary may not modify the proportion of African drones at the DCAs that virgin queens produced within that apiary may attend. Further research is needed to determine what concentration and distribution of managed European colonies are needed to modify the regional reproductive population to the extent that virgin queens produced in the apiaries providing drones would themselves attend a DCA with a high proportion of European drones. There likely is a balance between the number of European bee colonies needed in an area to saturate the regional DCAs and the resources available in the environment needed to sustain those colonies.

Past studies have shown a slight tendency towards like-subspecies matings where African and European queens mated with drones of their same subspecies 58% and 64% of the time, respectively [36]. Several theories, including partial physiological barriers, temporal isolation, or spatial isolation, exist to explain these tendencies [36–39]. In the present study, drones were collected from both 10 and 30 m elevations at each DCA to determine if there was a vertical distribution of African and European subspecies within the DCA. However, no detectable difference in vertical flight behavior of African and European drones was observed. This suggests that spatial isolation is not likely to be a contributing factor to any assortative mating that may be occurring.

The central Florida honey bee population offers a unique opportunity for understanding the interactions of African and European honey bee populations and the Africanization process. Furthermore, experimentation in this region promises to offer valuable insight into possible population manipulation techniques to limit the introgression of undesirable African honey bee genetics into the managed European honey bee population.

## Supporting Information

S1 Table Collection site, molecular profile, and maternal group assignment information for individual drones. Data are the name of the DCA in which the drones were caught (DCA ID), the distance of that DCA from a managed European honey bee apiary (DCA Location), the ID number of each drone (Bee ID), the fragment lengths of the allele present at each microsatellite loci (HB−C16−05, A107, B124, A79, A88, A7, HB−SEX−03, HB−SEX−01, HB−THE−03, and HB−THE−04), the haplotypes assigned based on the alleles present within the linkage groups (HB−SEX Haplotype and HB−THE Haplotype), the mitochondrial PCR−RFLP results (mtDNA, E = European, A = African), and the maternal group assignment made by COLONY based on the unlinked microsatellite fragment length and the linkage groups (COLONY Mother ID (by DCA ID)). Note that: 1) "**" denotes missing allelic information in allele and haplotype data columns. 2) Loci HB−SEX−03, HB−SEX−01, HB−THE−03, and HB−THE−04 are shaded grey to indicate that these data are not directly inputted into COLONY for maternal analysis due to linkage. Rather the linkage pairs are utilized to generate the haplotypes that are then used in the maternal analysis with the other unlinked loci. 3) Haplotype assignments and mother ID's are determined by DCA. Therefore, HB−SEX Haplotype (HB−THE Haplotype, or Mother ID) 1 at AK is not equivalent to HB−SEX Haplotype (HB−THE Haplotype, or Mother ID) 1 at WD (OSC, SIED, LBV, or MK).
(XLSX)
